# Riverine fish diversity varies according to geographical isolation and land use modification

**DOI:** 10.1002/ece3.3237

**Published:** 2017-08-30

**Authors:** Elizabeth Nicol, Jamie R. Stevens, Susan Jobling

**Affiliations:** ^1^ Department of Life Sciences Institute of Environment Health and Societies Brunel University Uxbridge Middlesex UK; ^2^ Department of Biosciences Geoffrey Pope Building University of Exeter Exeter UK

**Keywords:** biodiversity, fisheries, genetic diversity, land use, population ecology, species diversity

## Abstract

Understanding the environmental factors driving species‐genetic diversity correlations (SGDCs) is critical for designing appropriate conservation and management strategies to protect biodiversity. Yet, few studies have explored the impact of changing land use patterns on SGDCs specifically in aquatic communities. This study examined patterns of genetic diversity in roach (*Rutilus rutilus* L.) together with fish species composition across 19 locations in a large river catchment, spanning a gradient in land use. Our findings show significant correlations between some, but not all, species and genetic diversity end points. For example, genetic and species differentiation showed a weak but significant linear relationship across the Thames catchment, but additional diversity measures such as allelic richness and fish population abundance did not. Further examination of patterns in species and genetic diversity indicated that land use intensification has a modest effect on fish diversity compared to the combined influence of geographical isolation and land use intensification. These results indicate that environmental changes in riparian habitats have the potential to amplify shifts in the composition of stream fish communities in poorly connected river stretches. Conservation and management strategies for fish populations should, therefore, focus on enhancing connectivity between river stretches and limit conversion of nearby land to arable or urban use to maintain current levels of biodiversity.

## 1 INTRODUCTION

Species‐genetic diversity correlations (SGDCs) can reveal how factors of interest, including facets of environmental heterogeneity, influence ecological organization. To better evaluate the consequences of environmental threats, loss, and management of biodiversity, it is now possible to exploit the link between genetic diversity in populations of one species (the focal species) and the species diversity of the associated assemblage. Positive correlations between species and genetic diversity have been demonstrated across a variety of environments and species (including butterflies, Cleary et al., 2006; bats, Struebig et al., 2011; and fish, Blum et al., 2012) and are also supported by theoretical modeling of plant communities (Adams & Vellend, 2011). Conversely, an absence of coincident correlations has also been observed, sometimes in the same multispecies studies (Derry et al., 2009; Silvertown, Biss, & Freeland, 2009; Struebig et al., 2011) and at differing local, regional, and global scales (Taberlet et al., 2012; Vellend & Geber, 2005; Vellend et al., 2014). This lack of generality is a common pattern in ecology, where studies are often limited to a single measure of diversity. Combining multiple species measures at a variety of diversity levels can, therefore, better guide appropriate environmental and land use policies at the scale over which they are likely to be implemented (Donald, Sanderson, Burfield, & van Bommel, 2006).

Environmental characteristics are also known to influence patterns of species and genetic diversity, nonetheless whether simultaneous alterations depict a causal relationship that responds to site‐specific characteristics remains poorly explored (Vellend & Geber, 2005). This is especially true of aquatic systems, likely reflecting the difficulties of obtaining large‐scale datasets on diversity at both the assemblage and population‐genetic level. For example, Harding et al., (1998) demonstrate the detrimental influence of past and present day land use on current diversity in stream communities, and literature is extensive on the specific ecological impacts of eutrophication (Smith, 2003) on species diversity (Seehausen, van Alphen, & Witte, 1997; Vonlanthen et al., 2012). However, less is known about the implications for biodiversity of simultaneous changes in land use patterns within the proximity of rivers.

Large riverine catchments offer opportunities to explore the influence of environmental heterogeneity and connectivity on patterns of aquatic biodiversity. For example, individual dispersal in wild populations is frequently hampered by physical barriers or unsuitable habitat patches between suitable locales, enhancing divergence, and differentiation (Bergek & Olsson, 2009; Leclerc, Mailhot, Mingelbier, & Bernatchez, 2008; Ruzzante et al., 2006). Geographical isolation between locations within the dendritic structure of a river network has also been found to be largely deterministic of inter and intraspecific genetic diversity (Osborne et al., 1992). Aquatic populations living in close proximity are, therefore, assumed to exhibit higher levels of immigration that increase genetic diversity. In support of this assumption, Hamilton et al. (2014) demonstrated the existence of local subpopulations of roach within multiple locations throughout the UK at both the catchment and tributary scale, with populations showing evidence of restricted gene flow but not reproductive isolation. An isolation‐by‐distance pattern also indicated limited gene flow (<10 km) across the Thames catchment, a finding in agreement with a typical metapopulation structure within single drainages for this species. Thus, anthropogenic modifications to rivers, such as weirs and in‐stream impoundments, may impinge on population connectivity and isolate individuals, surreptitiously limiting genetic and species diversity.

River catchments such as the Thames are strongly influenced by their surroundings (Allan, 2004), and human‐induced modifications can alter living conditions for the aquatic organisms present. Thus, a transition from natural to intensively managed landscapes is likely to impact the integrity of resident fish populations, particularly when acting in concert with other environmental stressors (Acevedo‐Whitehouse & Duffus, 2009). Much of southeast England has been transformed from landscapes previously dominated by woodland and open grassland/floodplains, into large urban conurbations or high‐intensity agriculture. Such changes in land use practice have been linked to deterioration of biodiversity (Acevedo‐Whitehouse & Duffus, 2009; Bickham, Sandhu, Hebert, Chikhi, & Athwal, 2000; Frankham, Briscoe, & Ballou, 2002) and the declining abundance of aquatic populations (Harding et al., 1998). Testing both individual and combined environmental influences that shape fish populations will, therefore, provide a broader perspective on whether agricultural intensification and urbanization have been gained at the potential expense of riverine ecosystem health.

As such, the key objective of this study was to characterize the influence of proximal land use patterns and distance on both the genetic diversity of wild roach populations and the diversity of fish species assemblages throughout the Thames catchment. An increased presence of disturbed urban/arable land use was hypothesized to reduce both species and genetic diversity of stream fish within a river catchment, and a similar pattern was expected for increasing physical isolation between sample sites. In pursuit of this aim, key testable hypotheses allowed assessment of: (i) the extent to which the genetic and species structure of fish communities within the catchment were correlated; and (ii) the extent to which fluctuations in genetic and species diversity can be attributed to patterns of connectivity between localities, and, separately, to a gradient of watershed land use. Outcomes of this work will improve our understanding of the importance of environmental factors acting simultaneously to drive population success, thereby increasing our ability to improve fish population conservation and to mitigate the most influential factors impacting riverine communities.

## 2 METHODS

### 2.1 Study system

The Thames catchment is located in South East England and covers an area of approximately 16,000 km^2^, much of which is dominated by the capital city of London and associated urban conurbations. Consequently, the region is characterized by human‐modified landscapes, giving rise to an aquatic environment influenced by extensive urbanization and intensive agriculture practices. At the time of the last mapping exercise in 2004, 35% of the Thames Region was classified as arable, 19% grassland and 11% woodland, the rest being urban. The dominance of urban land is now likely to be significantly larger than in 2004 as the Thames catchment has undergone extensive human modification and management and, as a result, many contemporary riparian habitats within the catchment are expected to be influenced by both current and legacy effects of urbanization (Johnson et al., 2009).

### 2.2 Selection of the focal species, the roach, for genetic analyzes

The roach was chosen as the focal species as it is ubiquitous throughout English rivers and often dominates freshwater communities both numerically and in total fish biomass (Vollestad & Labeelund, 1987). Short spawning migrations and a retained dominance within stream communities (Labeelund & Vollestad, 1985) mean that roach can also exert strong effects on stream biota and primary productivity through competition and predation. Therefore, as a historically abundant and largely disturbance/pollution tolerant species, it can be reasoned that effects seen in this species may also be reflected in other more sensitive fish species.

### 2.3 Site selection

**Figure 1:**
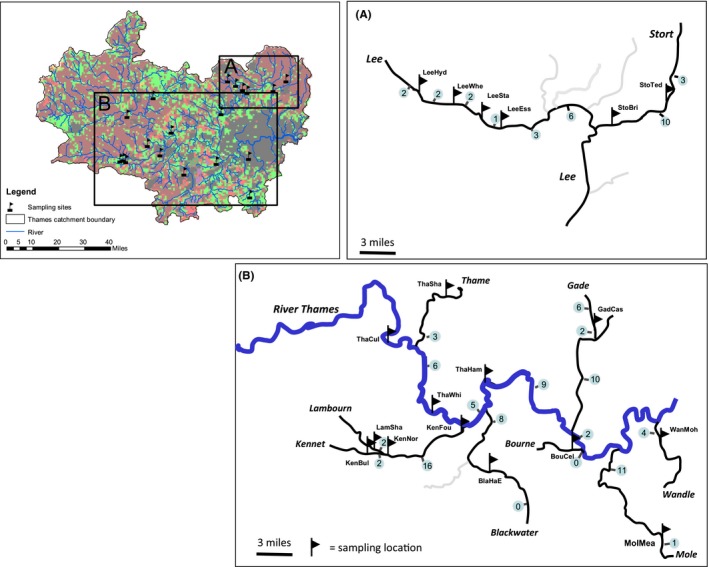
A) Locations of sample sites within the Thames catchment (marked by flags) and B) the number of physical barriers between sites (values in light blue circles)

In total, we analyzed population genetics, species composition, environmental, and land use data at 19 sites located across a number of sub‐basins within the larger Thames catchment (Figure 1; a subset of those used in Hamilton et al. (2014)). A single catchment was chosen in order to minimize the influence of underlying variation due to postglacial colonization and dispersal trajectories of roach among different UK river basins (Ketmaier, Bianco, & Durand, 2008; Larmuseau, Van Houdt, Guelinckx, Hellemans, & Volckaert, 2009). Sampling sites were chosen based on the likely presence of roach from historical records and encompassing sites representing the full span of agricultural and urban intensification identified using digitized Land Cover datasets (see Characterization of Environmental Variables; Land Use and Connectivity section for more details). In addition, sites were chosen where there was no recorded restocking, as this could have had significant implications for the genetic structure of roach populations. Restocking information was obtained for each of these sites from the Environment Agency Live Fish Movement Database (LFMD), which contains age, number, and weight of hatchery‐reared roach stocked in the Thames catchment from 2000 onwards. Despite the lack of long‐term (pre‐2000) restocking records, poststocking survival of stocked roach is estimated to be low, and approaches zero in certain circumstances (Aprahamian, Barnard, & Farooqi, 2004). Additionally, no genetic signatures of stocked roach were found in the Thames catchment in a previous study (Hamilton et al., 2014). Sampling locations were also identified on river stretches where movement of fish was restricted by the presence of in‐stream obstructions or migratory barriers. It was intended that the inclusion of such sites would allow the impact of potential barriers to fish movement (between upstream and downstream environments) on genetic/species‐environment patterns to be explored. Indeed, Hamilton et al. (2014) concluded that roach populations sampled from these Thames sites represented restricted subpopulations, exchanging a small number of individuals per generation – a finding which supports the assumption of limited dispersal of roach and suggests that local measures of genetic variation are indicative of the population living in a given stretch of water.

### 2.4 Sampling regime

All 19 locations were sampled during one season (2010) using 3‐pass catch depletion sampling of the total fish population (Environment Agency, 2016). Pulsed DC electrofishing was conducted at each sampling location to collect all individual fish within a 100 m reach (beginning at the sample grid reference and progressing downstream), restricted at both upstream and downstream extremities with large stop‐nets or physical barriers to fish movement. All fish captured were transferred to holding tanks, whilst three electrofishing passes were completed; fish were then identified to species level for species diversity estimates, measured to the nearest millimeter and then released. At the same time, non‐destructive samples of fin clips and scales were collected from adult roach (age 2 + , *n *=* *869) for genetic analysis. Fin clips were stored in 100% ethanol, and scales were kept dry in scale packets for subsequent DNA extraction. All animals used in this research were treated humanely and with regard to the alleviation of suffering; all procedures were subject to approval by the local ethical review process as required under the U.K. Animals (Scientific Procedures Act, 1986).

### 2.5 Genetic and species diversity calculations

Simple measures of total fish abundance, roach abundance, and species richness (i.e., number of different species caught) were calculated for every sample location. More complex indices of species diversity, including evenness (the relative abundance of rare and common species) and the Shannon (*H*) diversity index (which incorporates both species richness and evenness; Shannon & Weaver, 1949) were calculated to compare with genetic diversity measures.

Roach samples (*n *=* *869) were genotyped at 14 microsatellite loci and checked for null alleles and Hardy–Weinberg equilibrium, as described in Hamilton et al. (2014). Genetic diversity statistics (*H*_O_ and *AR*) for each roach population were taken from Hamilton et al. (2014). In addition, the individual genetic diversity of each roach, which was calculated in this study using internal relatedness (*IR*), a multilocus measure of relatedness and inbreeding. *IR* estimates the similarity between parental half‐genotypes within an individual and weights the importance of each allele according to its frequency in the population (Amos et al., 2001). *IR* has previously been shown to negatively influence reproductive success in a wide range of aquatic organisms, such as Atlantic salmon (*S. salar,* Garant, Dodson, & Bernatchez, 2005), the long‐finned pilot whale (*Globicephala melas*) and gray seal (*Halichoerus grypus*, both Amos et al., 2001), so was examined here to obtain information on roach population fitness. Internal relatedness was calculated using an R extension package (*Rhh*; available at http://www.helsinki.fi/biosci/egru/research/software); mean *IR* was then calculated for each site by averaging *IR* from all individuals. The *Rhh* package also creates outputs of homozygosity by locus (*HL*, Aparicio, Ortego, & Cordero, 2006) and standardized heterozygosity (*SL*); we present only results for *IR* here, as *IR*,* SL,* and *HL* were highly correlated (*r *=* *.98, *p *=* *.001).

### 2.6 Characterization of environmental variables; land use and connectivity

Here, we define environmental variation as conditions attributable to watershed land use/cover. Local (2 km) landscape composition was assessed at each sample site from a digital land cover map (LCM2007). This map is derived from categorized parcels of satellite images, projected in ArcGIS 10 (ESRI 2011, Redlands, CA: Environmental Systems Research Institute). The LCM2007 25 m raster dataset (distributed by CEH Information Gateway, Wallingford, UK, 2011) provides digital cartography of the UK, defining broad habitats seen in the UK Biodiversity Action Plan (UK BAP). LCM2007 is the most up to date digital map overlay available, however, it is appropriate to be mindful of changes to land use that may have occurred in the interim, which could alter river habitats over more recent timescales.

For the purpose of this work, an assessment of landscape characteristics within a circular buffer zone of 2 km surrounding each sampling site were recorded as the proportion of arable, grass, wood, and urban land (Table 1). A 2 km buffer zone was chosen because, based on previous studies, roach are likely to complete their full life cycle in <2.5 km (Geeraerts et al., 2007), so this area was deemed likely to encompass the local land use that impacts directly on the stretch of river commonly used by roach at a given sampling site. The Spatial Analyst toolbox in ArcGIS10 was used to calculate the relative percentage of each land use type within the associated buffer area, and the dominant land use class was determined as the category contributing the highest percentage within the 2 km buffer area (Table 1). Land use percentages were then used in subsequent analyses, as well as UK BAP categories of dominant land use seen at each site (e.g., arable, woodland, grassland, and urban). Land use percentages from 2 km buffers were also summed to form surrogate variables of “disturbed” (arable + urban) and “natural” (grassland + woodland) land at each sample site. This concept has been used previously, where a summary statistic of “disturbance” was demonstrated to be more indicative of degradation in stream ecosystems and a reliable proxy for human activity (Allan, 2004).

**Table 1:** Dominant land use class (a) and the percentage of each class constituting 2 km buffer zones (b) surrounding each site sampled across the Thames catchment

	Woodland	Arable	Improved Grassland	Urban
a) Dominant land use class				
Number of sites	0	6	6	7
b) % Land use				
Median	0.2	18.5	22.5	19
Range	0–19	0–90	0–84	0–100

The movement of fish between sites and the physical carrying capacity of each location can determine the number of individuals and species present. Accordingly, the waterway distance of each sample site from the main stem Thames (km) was included as an additional covariate when comparing species diversity and roach genetic diversity between sites. Measurement of pairwise waterway distance between sites (km) was calculated via ArcMap 10 (ESRI, Redlands, CA, USA) using the Fastest Path routine in conjunction with Network Analyst. The same approach was used to calculate waterway distances from each sample location to the main river. Pairwise waterway distance and distance to the main stem Thames were used to examine the role of isolation from the main migration corridor and to define the influence of watershed location within the Thames on the basis of genetic and species assemblage similarity.

### 2.7 Statistical analysis of environmental variation, genetic diversity, and species diversity

Pearson correlation coefficients were used to examine relationships between roach abundance, the genetic diversity of roach populations and overall species diversity across all sites. More specifically, measures of mean population internal relatedness and genetic diversity (*IR, AR,* and *H*_O_), abundance and species diversity of the fish assemblage (Shannon diversity, evenness, and species richness) were compared to assess the strength of pairwise association between indices. Partial Pearson correlations accounting for distance from the main stem Thames were also conducted to examine if species‐genetic diversity relationships changed when the influence of isolating distance was removed.

Pairwise Bray–Curtis dissimilarity in species composition of fish assemblages between sites was calculated using multidimensional scaling (MDS) as implemented in SAS 9.3 (SAS Institute Cary, NC). Mantel tests (using GenAlEx 6.5) were used to compare pairwise estimates of geographic distance to estimates of species assemblage dissimilarity between all 19 sites, to evaluate the strength of biogeographical structuring. Mantel tests were also conducted to assess the strength of association between pairwise estimates of assemblage differentiation (Bray–Curtis dissimilarity) and genetic differentiation (linearized *F*_ST_ = *F*_ST/_ *1‐F*_ST_) among localities and in relation to the number of physical barriers between sites.

Differences in genetic and species diversity of fish populations were compared across categories of dominant land use using ANOVA. Linear regressions and correlations performed in SPSS were used to examine relationships between land use percentages and (i) species diversity (*H*), abundance and species richness; (ii) allelic richness (*AR*), genetic diversity (*H*_O_), and mean internal relatedness (*IR*) of roach populations. Geographic distance from the main stem Thames was also included in regression analyzes as a covariate alongside “disturbed” land use percentages. All test variables used were inspected prior to analysis for normality using the Kolmogorov–Smirnov (K–S) test and, if requirements of a normal distribution were not met, data were log‐transformed. α = 0.05 was chosen as the accepted significance level for all statistical tests. Unless otherwise specified, statistical analyses were carried out with SPSS, Ver. 20 (IBM Chicago, IL).

## 3 RESULTS

### 3.1 Population‐genetic structure of roach and relationship to species diversity

In total, genetic data were obtained for 869 individual roach from 19 sites in the Thames catchment, encompassing a gradient of land use and covering a riverine distance of 176 km (out of 354 km from source to final outlet into the southern North Sea). Genotype data revealed high genetic diversity in all roach populations examined across the Thames catchment (Table 2). When testing relationships among various measures of genetic diversity (of roach) and species diversity indices (calculated from the overall stream fish assemblage), none showed a significant correlation (Table 3, Bonferroni corrected *p‐*values). Only paired genetic diversity measures (*IR* vs *H*_O_) and species diversity (*H* vs Evenness, Abundance, and Roach abundance) showed a significant linear increase in relation to each other across all correlation analyses conducted.

**Table 2:** Summary of species and genetic diversity end points calculated for each of the 19 sample sites within the Thames catchment. All genetic diversity measures are averages for roach sampled at each site

	River	Site name	Assemblage endpoints					Genetic diversity measures		
			Species richness	Shannon diversity (*H*)	Evenness	Fish abundance	Roach abundance	*Mean IR*	*H*_O_	*AR*
1	Blackwater	Hawley meadows	7	1.13	0.58	555	322	0.036	0.70	7.37
2	Bourne	Chertsey	8	1.58	0.76	154	73	0.024	0.74	8.35
3	Gade	Cassiobury Park	9	1.65	0.75	530	85	0.031	0.73	8.11
4	Kennet	Bulls lock	9	1.66	0.76	114	41	0.044	0.74	8.44
5	Kennet	Foundry brook	8	1.49	0.72	119	60	0.015	0.75	8.71
6	Kennet	Northcroft	9	1.14	0.52	287	180	0.031	0.73	8.47
7	Lambourn	Shaw	4	1.06	0.76	77	39	0.063	0.72	6.79
8	Lea	Essendon	8	0.93	0.44	409	297	0.080	0.69	8.29
9	Lea	Hyde Mill	6	0.77	0.43	43	1	0.120	0.68	8.17
10	Lea	Stanborough	7	1.43	0.74	335	56	0.030	0.73	8.37
11	Lea	Wheathampstead	6	1.48	0.83	723	173	0.056	0.72	8.46
12	Mole	Meath Green	8	1.35	0.65	943	467	0.005	0.75	8.42
13	Stort	Briggens	9	1.63	0.74	204	89	0.104	0.67	8.07
14	Stort	Tednambury	9	1.06	0.48	410	285	0.074	0.69	8.21
15	Thames	Culham	11	1.34	0.56	307	32	0.043	0.73	8.76
16	Thames	Hambledon	9	1.58	0.72	127	77	0.033	0.73	8.23
17	Thames	Shabbingdon	11	1.60	0.67	729	293	0.002	0.75	7.93
18	Thames	Whitchurch	9	0.98	0.45	298	113	0.027	0.73	8.22
19	Wandle	Morden Hall	6	1.36	0.76	229	25	0.022	0.75	8.20

**Table 3:** Correlation between species diversity and roach genetic diversity measures

		*H*	Species richness	Abundance	Roach abundance	Evenness	*Mean IR*	*H*_O_	*AR*
*H*	Correlation	1	0.479	0.167	−0.069	0.838**	−0.467	0.406	0.028
	Sig. (2‐tailed)		0.038	0.495	0.778	<0.001	0.044	0.084	0.911
Species richness	Correlation		1	0.251	0.322	−0.059	−0.298	0.106	0.319
	Sig. (2‐tailed)			0.300	0.178	0.810	0.216	0.667	0.184
Abundance	Correlation			1	0.823**	0.054	−0.372	0.237	0.186
	Sig. (2‐tailed)				<0.001	0.825	0.117	0.329	0.445
Roach abundance	Correlation				1	−0.239	−0.228	0.038	0.140
	Sig. (2‐tailed)					0.325	0.347	0.878	0.567
Evenness	Correlation					1	−0.364	0.403	−0.263
	Sig. (2‐tailed)						0.125	0.087	0.276
*Mean IR*	Correlation						1	−0.944**	−0.234
	Sig. (2‐tailed)							<0.001	0.334
*H*_O_	Correlation							1	0.185
	Sig. (2‐tailed)								0.449
*AR*	Correlation								1
	Sig. (2‐tailed)								

*N* = 19 for all variables. **Correlation is significant at the Bonferroni corrected *p* value of .0014.

### 3.2 Fish species patterns across the catchment

Catch data from routine surveys of 19 Thames catchment sites allowed examination of fish population composition in parallel with genetic diversity of roach populations. Sixteen freshwater fish species were commonly recorded throughout the Thames catchment in various proportions at each site, with roach being the most abundant species encountered (Table S2; total number = 2,959, 43.9% of total catch of all species). Other cyprinids were also ubiquitous across all sites sampled; cyprinids in general made up over 80% of the total catch across all sites (roach> chub> gudgeon> dace), followed by the most prevalent non‐cyprinid species, perch (532 fish, 7.9% of the total catch). Total abundance of fish caught at each site varied by more than a factor of 10 across the catchment (Table 2); the highest at Meath Green (River Mole) and the lowest at Hyde Mill (River Lea) (with counts of 943 and 43, respectively). These same sites also had the highest and lowest roach abundance, respectively. Intersite comparisons of assemblage composition demonstrated a range of species richness values between 4 and 11 species, with associated Shannon diversity indices ranging between 0.77 and 1.66 across the 19 sites (Table 2). Interestingly, the most and least evenly distributed communities were found to be sites on the same tributary, Wheathampstead (0.83) and Hyde Mill (0.43) on the River Lea, respectively.

### 3.3 Role of isolation in driving patterns of fish diversity

**Figure 2:**
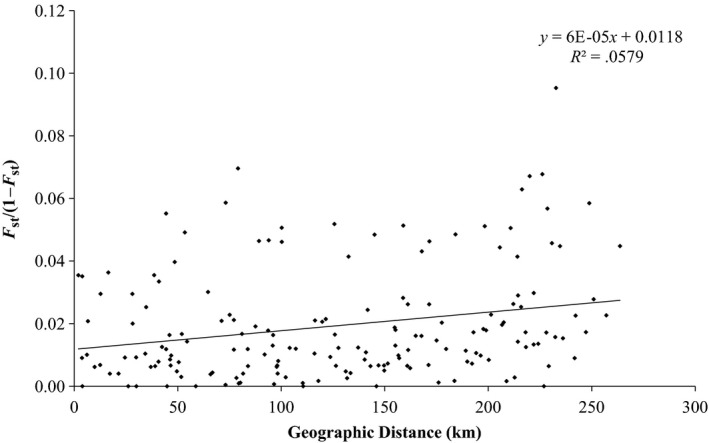
Comparison of geographic distance to linearized *F*_ST_ genetic distances across all sites, with regression line.

**Figure 3:**
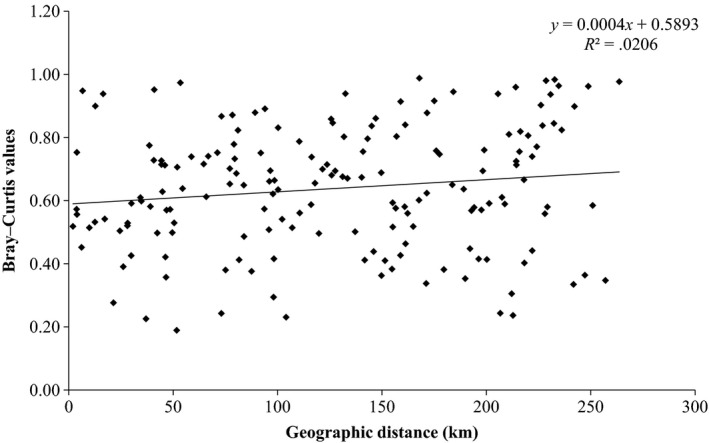
Comparison of geographic distances between sampling sites to Bray–Curtis values of species dissimilarity across all 19 sites with regression line.

**Figure 4:**
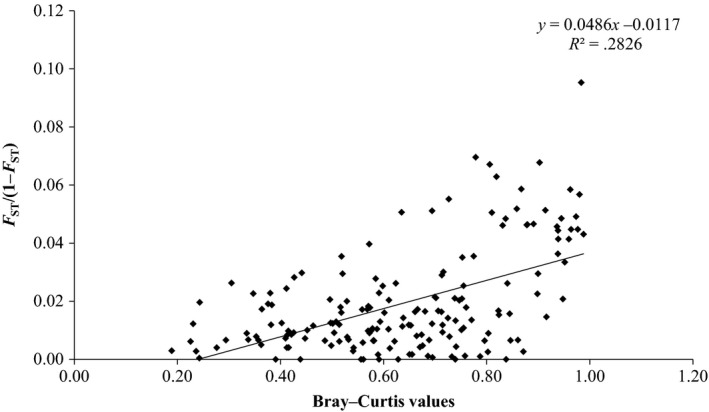
Comparison of Bray–Curtis distances to linearized *F*_ST_ genetic distances across all 19 sites, with regression line.

Accounting for connectivity between sample sites is of crucial importance when examining spatial patterns of species and genetic diversity. The role of distance in driving patterns of genetic and species differentiation was, therefore, examined to see if evidence of limited dispersal was present in the Thames and, overall, increasing isolation between sample sites appeared to promote both assemblage and genetic differentiation. Here, pairwise linearized *F*_ST_ (Slatkin, 1985) of 19 sites in the Thames catchment showed a weak but significant relationship with riverine distance (Figure 2; *r* ^*2*^ **=* *.057, *p *=* *.01), which was also evident when testing assemblage divergence of stream fishes across the Thames catchment (Bray–Curtis dissimilarity values vs riverine distance; Figure 3; *r* ^*2*^ **=* *.02, *p *=* *.05). Our results indicated a strong relationship between population‐genetic divergence (linearized *F*_*ST*_) and fish assemblage divergence (Bray–Curtis distance, Figure 4; *r* ^*2*^ **=* *.28, *p *=* *.01), which was not evident when examining simple correlations between species and genetic diversity measures (Table 3). In order to better understand the influence of physical barriers on fish assemblage structure and roach genetic variation, enumeration of the number of barriers between sample sites was also tested against pairwise roach population‐genetic divergence (linearized *F*_*ST*_) and fish assemblage divergence (Bray–Curtis distance). Statistical correlation between pairwise matrices was significant only when examining linearized *F*_*ST*_ (*r* ^*2*^ **=* *.15, *p *=* *.01) in relation to the number of physical barriers between sample sites within the Thames catchment. This suggests increasing genetic divergence between sites with a larger number of physical barriers between them – a relationship that was not replicated at the species (diversity) level.

### 3.4 Response of fish diversity to changing land use patterns

This study indicates that sample sites dominated by urban development or grassland demonstrated no significant differences in mean *H*,* H*_O,_ or *IR* (Figure S1A and B). Instead, fish populations found at sites surrounded by predominantly arable land consistently demonstrated significantly lower mean *H*,* H*_O_, or higher *IR* (more inbred) when compared to grassland populations, a finding which agrees with the results shown in Table 4. Further examination of population data also suggests that, overall, sites dominated by grassland had larger fish populations with a greater abundance of roach. Together, these results suggest a negative influence of arable land on the diversity of fish populations (compared to those surrounded by grassland), a pattern which was also evident between *H*,* H*_O_, and *IR* when controlling for downstream distance to the main stem Thames (Table 4).

**Table 4:** Correlation values between land use (%) and diversity measures, controlling for downstream distance

		Abundance	*H*	Roach abundance	*Mean IR*	*H*_O_
Woodland	Correlation	−0.107	0.178	−0.062	0.025	0.024
	Significance (2‐tailed)	0.672	0.481	0.807	0.922	0.924
Arable	Correlation	−0.340	−0.496	−0.252	0.624	−0.658
	Significance (2‐tailed)	0.168	0.036	0.314	0.006	0.003
Grassland	Correlation	0.601	0.409	0.536	−0.426	0.270
	Significance (2‐tailed)	0.008	0.092	0.022	0.078	0.278
Urban	Correlation	−0.172	0.006	−0.205	−0.166	0.295
	Significance (2‐tailed)	0.496	0.980	0.415	0.511	0.234

### 3.5 Fish population response to disturbed land use and isolation

Testing species and genetic diversity patterns in relation to combined urban and arable (disturbed) land use practices showed a negative influence of % disturbed land on aquatic biota (although none were statistically significant; Table 5). However, further analysis showed that when % disturbed land and distance from the main stem Thames (geographic connectivity) were included as interacting factors in a linear regression model, they became a significant collective predictor of four key diversity indices (*H*, Species richness, *IR,* and *H*_O_; Table 5). Fish populations in the Thames catchment that are increasingly isolated from the main stem river and surrounded by more disturbed land exhibit lower species richness/diversity, decreased genetic diversity (*H*_O_) and increased inbreeding (i.e., higher *IR*). However, this interaction between % disturbed land and distance from the main stem Thames was not a significant predictor of *AR*, roach abundance or total abundance of fish assemblage.

**Table 5:** Statistical regression relationships between different measures of diversity and predictor variables, including disturbed land use (%), distance downstream to the main stem River Thames (km) and both distance and disturbed land use in combination

Independent variable	Dependent variable	Test statistic	Significance
Disturbed land (%)	*H*	*r* ^2^ * *=* *.145	.098
	Species richness	*r* ^*2*^ * *=* *.196	.051
	Abundance	*r* ^*2*^ * *=* *.104	.165
	Roach Abundance	*r* ^2^ * *=* *.356	**.034**
	*Mean IR*	*r* ^*2*^ * *=* *.180	.062
	*H*_O_	*r* ^2^ * *=* *.166	.074
	AR	*r* ^2^ * *=* *.067	.270
Distance to main stem Thames (km)	*H*	*r* ^2^ * *=* *.099	.176
	Species richness	*r* ^2^ * *=* *.138	.107
	Abundance	*r* ^2^ * *=* *.153	.088
	Roach Abundance	*r* ^2^ * *=* *.356	**.043**
	*Mean IR*	*r* ^2^ * *=* *.243	**.027**
	*H*_O_	*r* ^2^ * *=* *.234	**.031**
	*AR*	*r* ^2^ * *=* *.013	.632
Disturbed land * Distance to main stem Thames	*H*	*r* ^2^ * *=* *.255	**.023**
	Species richness	*r* ^2^ * *=* *.226	**.034**
	Abundance	*r* ^2^ * *=* *.000	.962
	Roach Abundance	*r* ^2^ * *=* *.001	.924
	*Mean IR*	*r* ^2^ * *=* *.482	**.001**
	*H*_O_	*r* ^2^ * *=* *.441	**.002**
	*AR*	*r* ^2^ * *=* *.068	.265

H, Shannon diversity; IR, internal relatedness; H_O_, heterozygosity; AR, allelic richness.

Significant values are highlighted in bold.

## 4 DISCUSSION

Understanding the principal factors driving population success is fundamental to the conservation of natural resources (Frankham et al., 2002). However, detailed patterns of variation in species and genetic diversity are not often considered in parallel with respect to environmental conditions. Here, we demonstrate that environmental changes in riparian habitats are amplifying shifts in the composition and integrity of stream fish communities in poorly connected river stretches. Patterns of species diversity and genetic diversity across fish populations in the Thames reflect a suite of dynamic attributes that are likely to fluctuate under disturbance regimes driven by changing land use patterns across the catchment. Further interpretation of our results suggests that an increasing prevalence of arable/urban land and geographic isolation, act in concert to negatively impact both species diversity and genetic diversity of stream fishes. Conservation practices maintaining grassland in the area surrounding river stretches and actions that enhance connectivity between localities may, therefore, actively protect both species and intra‐population‐genetic diversity and are widely encouraged on the basis of these findings.

### 4.1 Relationships between fish species diversity and genetic diversity of roach

Positive relationships between species diversity and genetic diversity have been found in a range of other wild species and environments (Vellend, 2003; Vellend & Geber, 2005) including organisms such as insects (Papadopoulou et al., 2011), butterflies (Cleary et al., 2006), zooplankton (Derry et al., 2009), bats (Struebig et al., 2011), and stream fishes (Blum et al., 2012). Indeed, Blum et al. (2012) found a positive relationship between stream fish assemblage diversity and allelic richness of central stonerollers (*Campostoma anomalum*). In our study, only genetic and species assemblage divergences appear to covary across the Thames catchment (*F*_*ST*_ vs Bray–Curtis *p *=* *.01) suggesting inconsistent patterns between the different measures of diversity used. A plausible explanation for the lack of symmetry in diversity responses is that the genetic diversity of roach was strongly driven by population size at the time of sampling, whereas Shannon diversity was less affected by changes in roach abundance, a similar result to that reported by Blum et al. (2012). Changes in abundance of roach, therefore, exert a greater effect on roach genetic diversity than on habitat‐specific diversity (*H*), because smaller roach populations allow greater overall species diversity and richness. Genetic diversity of an individual species is likely to be more affected by fluctuations in abundance observed across sampling sites, altering genetic variation through the presence/absence of different alleles under different population size scenarios (Frankham et al., 2002). Our findings support this theory, suggesting that the genetic diversity measures of *IR* and heterozygosity in roach are sensitive to the abundance of roach and to the size of the total fish assemblage when controlling for downstream distance to the main river stem (*IR*,* p *=* *.001 and *H*_O_, *p *=* *.016), but this change in abundance does not negatively impact species diversity indices. Genetic diversity of roach populations fell with declining population size, (a finding paralleled by little change in species diversity) due to the removal of few individuals from an abundant species, which appears to have only minimally affected assemblage structure. This is likely due to the high level of competition for resources in the Thames, such that when a species or a number of individuals are removed from a population, another species/individual flourishes and replaces them quickly.

### 4.2 Influence of isolation on fish populations

River networks offer an opportunity to study the influence of landscape and proximal environmental attributes on diversity, as organisms are intrinsically tied to their geomorphological landscape. Many of the cases reported in the literature which document positive patterns between species and genetic diversity have been conducted in isolated situations where the number of species and genetic diversity will be naturally reduced at a small geographic scale (Struebig et al., 2011; Vellend, 2003). Our results show that even in a relatively large catchment, isolation from dispersal corridors can drive assemblage and population composition in fish species. Longitudinal distance between sample sites and the placement of sample sites across a drainage network has previously been found to influence patterns of genetic diversity in stream fishes (Costello, Down, Pollard, Pacas, & Taylor, 2003) and the robust correlations observed in our study uphold theoretical expectations of genetic and assemblage divergence, varying in parallel with geographic isolation (Vellend & Geber, 2005).

Furthermore, patterns of connectivity and isolation between sample sites influenced genetic profiles among roach populations more than overall fish species diversity. The decay in genetic similarity with distance has been associated with decreasing similarity in environmental landscapes or due to dispersal barriers between locales, limiting drift and migration (Sei, Lang, & Berg, 2009; Soininen, McDonald, & Hillebrand, 2007). The number of in‐stream barriers, of both man‐made and natural origin can compound physical isolation between sites (Crispo, Bentzen, Reznick, Kinnison, & Hendry, 2006; Hanfling, Durka, & Brandl, 2004), and this was shown to be the case when examining genetic divergence between sites within the Thames catchment. Conducting a similar study with a species with more restricted movement (*Gobidae*) or more sensitive to environmental degradation (*Salmonidae*) would perhaps offer further insight into the relative importance of connectivity or habitat heterogeneity as the most important deterministic features shaping other species in stream fish assemblages within the Thames. For example, multispecies studies – which collect species data and genetic data from multiple species in the same system (Lamy et al., 2013; Robinson et al., 2010; Wei & Zhang, 2010) – could be employed to indicate the species most affected by selective and nonselective factors, giving a better indication of the generality of correlation tendencies between species and genetic data.

### 4.3 Environmental predictors of species and genetic diversity

In this study, we utilized the summary statistic of “disturbed” land use (combining urban and arable land) as a proxy of human activity – an approach that has previously been used elsewhere as a surrogate for degradation in stream ecosystems (Allan, 2004). Exploiting the natural dendritic structure of tributaries within a single catchment demonstrated that genetic diversity and species diversity are lower in isolated environments surrounded by disturbed land. The precise environmental driver that gives rise to this pattern is unknown but previous studies have shown that human alteration of catchment land use can adversely affect water quality and degrade stream channels (Diana, Allan, & Infante, 2006); in turn, flow modifications and poor habitat can also lead to significant losses in diversity and reduced abundance of stream fish (Roy et al., 2005; Silbiger et al., 2001). Overall, parallel effects of land use intensification, largely through conversion of land which was previously dominated by woodland or grassland, resulted in a negative influence on species and genetic diversity of fish across the study area, indicating a significant detrimental effect of disturbed land use on local riverine habitat.

Patterns of genetic diversity have previously been linked to habitat quality (Waits, Bagley, Blum, McCormick, & Lazorchak, 2008) and environmental heterogeneity (Bagley, Jackson, Franson, & Waits, 2004). When combining isolation distance from the main stem Thames and the amount of disturbed land within 2 km of the sample site, we showed that measures of genetic diversity are reduced significantly in isolated and highly disturbed environments. Similarly, average internal relatedness of roach populations increased significantly (indicating more inbreeding) with the combination of increasing isolation from the main stem Thames and land use disturbance (*IR*:* p *=* *.001). Previous declines in genetic diversity of stream fishes have been linked to intensive land use patterns (Blum et al., 2012) – a pattern also observed in some other taxa – for example, the genetic diversity of populations of *Daphnia magna* was found to be negatively impacted by agricultural land use intensity (Coors, Vanoverbeke, De Bie, & De Meester, 2009). Thus, we can conclude that disturbed landscapes are likely to drive degradation in proximal stream habitats, by lowering genetic and species diversity and increasing inbreeding in geographically isolated fish populations, which are likely to exchange only a limited number of individuals. From a conservation perspective, if we are to limit the detrimental impact of high‐intensity land use practices on riverine biota, it is therefore essential to enhance connectivity, to promote high levels of diversity in stream fishes, thereby limiting further effects of environmental degradation (Kahilainen, Puurtinen, & Kotiaho, 2014).

### 4.4 Implications for future research

The sustained presence of roach and also a higher mean *IR* in isolated/degraded river stretches reinforces their apparent tolerance to habitat disturbance in comparison with other freshwater fish species. By way of example, this study documents high levels of genetic diversity in many roach populations in the study area, whereas some sites within the Thames catchment exhibit low fish species richness and assemblage diversity. Thus, we recognize that further testing of our findings across different river catchments and exploration of patterns of genetic diversity within other focal species could prove highly insightful. Comparisons of genetic diversity in fish species that are less tolerant or less vagile than roach may be more indicative of the risk posed by habitat disturbance or fragmentation (Struebig et al., 2011) to the wider fish community. Future research should focus on a multispecies approach, analyzing genetic diversity in more than one species within an assemblage, which will add to our understanding of mechanisms underlying assemblage and population success (Taberlet et al., 2012).

## CONFLICT OF INTEREST

None declared.

## DATA ACCESSIBILITY

Microsatellite genotypes: see supplementary file in Hamilton et al., 2014.

## AUTHOR CONTRIBUTIONS

EN participated in the design of the study, collected field data, carried out the statistical analyzes, and drafted the manuscript. SJ and JS helped conceive and draft the manuscript. All authors gave final approval for publication.

## Supporting information


** **

** **
