# Supplementary material for: Riverine fish diversity varies according to geographical isolation and land use modification
Source: Ecol Evol. 2017 Aug 30;7(19):7872–83. doi: 10.1002/ece3.3237 (PMC5632612; doi:10.1002/ece3.3237)
Supplement: Supplementary file 1 [file ECE3-7-7872-s001.docx]

**km LCM category**

**2**

Grassland

Urban

Arable

**Genetic Diversity (H_O_)**

0.76

0.74

0.72

0.70

0.68

**Shannon Diversity (H)**

2.00

1.80

1.60

1.40

1.20

1.00

A

Error Bars: +/- 1 SE

Figure S1 (A) Patterns of mean species and genetic diversity in response to land use categories present across all sample sites within the Thames catchment. Squares are mean Shannon diversity (*H*) and triangles are mean genetic diversity (*H*_O_) across land use categories.


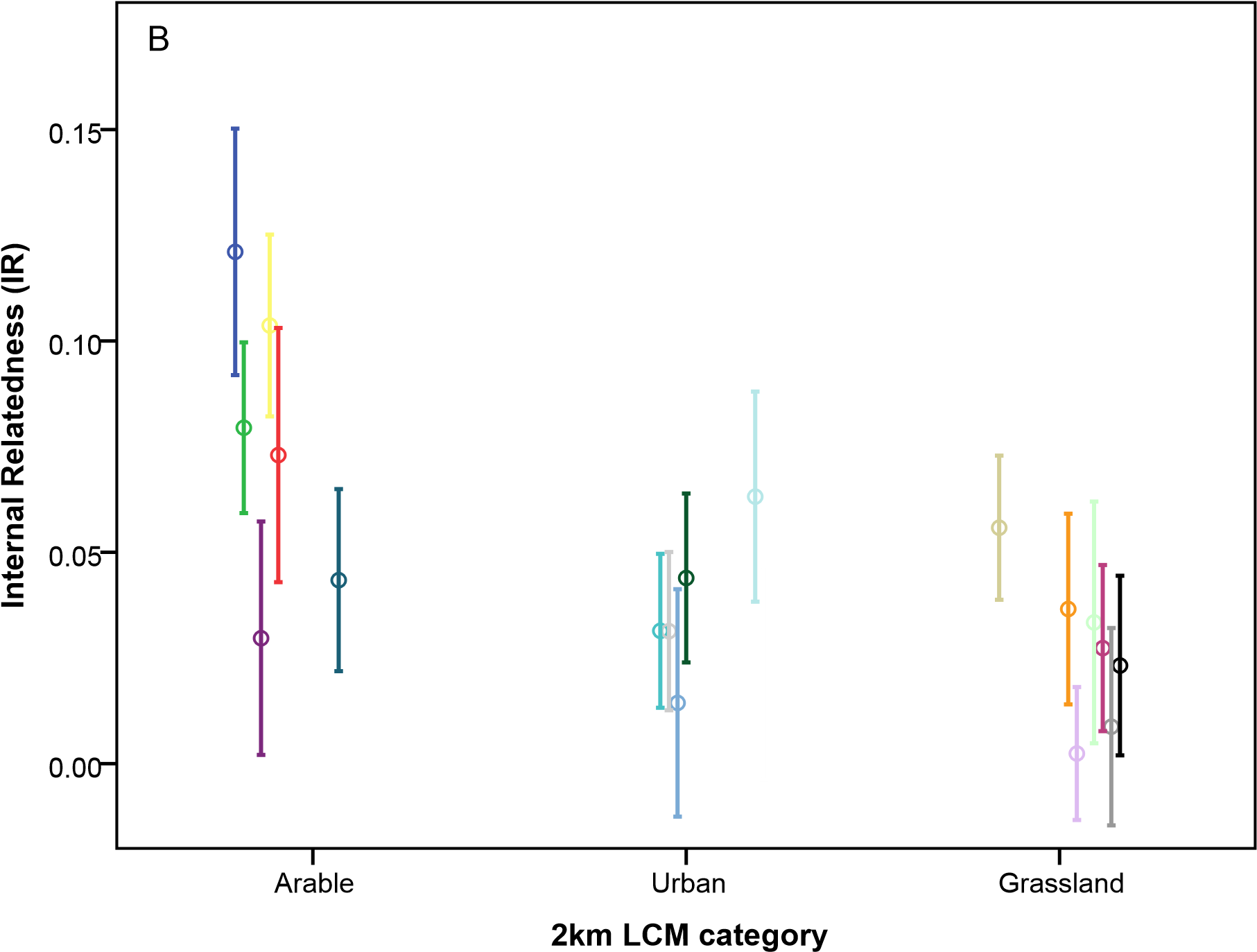


Error Bars: +/- 1 SE

Figure S1 (B) Patterns of mean *IR* across land use categories present across each sample sites within the Thames catchment. Open circles represent mean *IR* values, derived from all individuals at each of the 19 sample sites. Coloured bars represent different sites.
