# Supplementary material for: Riverine fish diversity varies according to geographical isolation and land use modification
Source: Ecol Evol. 2017 Aug 30;7(19):7872–83. doi: 10.1002/ece3.3237 (PMC5632612; doi:10.1002/ece3.3237)
Supplement: Supplementary file 2 [file ECE3-7-7872-s002.docx]

Appendix

| **Site Names** | **Latitude** | **Longitude** | **River width (m)** | **2km Woodland (%)** | **2km Arable (%)** | **2km Improved grassland (%)** | **2km Urban (%)** | **% Natural land** | **% Disturbed land** | **2km LCM category** | **Approx. dist to mainstream Thames (km)** | **Number of roach sampled (for genetics)** |
| --- | --- | --- | --- | --- | --- | --- | --- | --- | --- | --- | --- | --- |
| **BlaHaw** | 51.32411914 | -0.766560584 | 6 | 10 | 0 | 5 | 85 | 15 | 85 | 10 | 32 | 25 |
| **BouCeL** | 51.380862 | -0.477114 | 6 | 7 | 4 | 49 | 40 | 56 | 44 | 4 | 2 | 56 |
| **GadCas** | 51.65893149 | -0.425588116 | 6 | 18 | 0 | 10 | 72 | 28 | 72 | 10 | 31 | 56 |
| **KenBul** | 51.39707172 | -1.28485322 | 15 | 19 | 7 | 20 | 41 | 39 | 48 | 10 | 27 | 51 |
| **KenFou** | 51.43563926 | -0.976635218 | 16 | 0 | 6 | 18 | 76 | 18 | 82 | 10 | 4 | 32 |
| **KenNor** | 51.40165014 | -1.337252855 | 15 | 0 | 17 | 25 | 58 | 25 | 75 | 10 | 31 | 52 |
| **LamSha** | 51.40816479 | -1.308428 | 11 | 6 | 18 | 5 | 71 | 11 | 89 | 10 | 30 | 41 |
| **LeaEss** | 51.77279275 | -0.188182443 | 11 | 16 | 35 | 28 | 21 | 44 | 56 | 3 | 52 | 56 |
| **LeaHyde** | 51.83957549 | -0.358254611 | 6 | 0.4 | 77 | 5 | 17 | 5.4 | 94 | 3 | 63 | 28 |
| **LeaSta** | 51.78939999 | -0.224960893 | 8 | 4 | 40 | 18 | 38 | 22 | 78 | 3 | 56 | 31 |
| **LeaWhe** | 51.81423968 | -0.289033502 | 7 | 0 | 45 | 48 | 8 | 48 | 53 | 4 | 62 | 55 |
| **MolMea** | 51.19027737 | -0.185812041 | 8 | 0 | 10 | 84 | 6 | 84 | 16 | 4 | 51 | 42 |
| **StoBri** | 51.77989064 | 0.050242469 | 10 | 0 | 58 | 32 | 10 | 32 | 68 | 3 | 35 | 52 |
| **StoTed** | 51.831149 | 0.168921 | 9 | 0 | 90 | 6 | 4 | 6 | 94 | 3 | 47 | 30 |
| **ThaCul** | 51.65046248 | -1.267385334 | 34 | 0 | 77 | 19 | 4 | 19 | 81 | 3 | 0 | 44 |
| **ThaHam** | 51.55989314 | -0.873465389 | 64 | 13 | 28 | 59 | 0 | 72 | 28 | 4 | 0 | 44 |
| **ThaSha** | 51.75280736 | -1.033189595 | 10 | 0 | 19 | 81 | 0 | 81 | 19 | 4 | 25 | 50 |
| **ThaWhi** | 51.48661695 | 1.08974 | 48 | 0 | 22 | 73 | 5 | 73 | 27 | 4 | 0 | 60 |
| **WanMoh** | 51.403288 | 0.188209 | 11 | 0 | 0 | 0 | 100 | 0 | 100 | 10 | 7 | 48 |

Table S1. Additional variables used in correlation analyses conducted across 19 sites in the Thames catchment

|  | **Total number caught across all sampling sites** |
| --- | --- |
| ***Roach x bream hybrid*** | 4 |
| ***Common carp [Cyprinus carpio]*** | 6 |
| ***Brown trout [Salmo trutta]*** | 7 |
| ***Rudd varieties [Scardinius erythrophthalmus]*** | 10 |
| ***Tench [Tinca tinca]*** | 13 |
| ***Grayling [Thymallus thymallus]*** | 28 |
| ***Common bream [Abramis brama]*** | 38 |
| ***European eels [Anguilla anguilla]*** | 62 |
| ***Pike varieties [Esox lucius]*** | 108 |
| ***Barbel [Barbus barbus]*** | 122 |
| ***Bleak [Alburnus alburnus]*** | 376 |
| ***Perch [Perca fluviatilis]*** | 532 |
| ***Dace [Leuciscus leuciscus]*** | 672 |
| ***Gudgeon [Gobio gobio]*** | 708 |
| ***Chub [Leuciscus cephalus]*** | 1101 |
| ***Roach [Rutilus rutilus]*** | 2959 |
| Grand Total | 6746 |

Table S2. Total catch data used in species diversity calculation
